# From research to rapid response: mass COVID-19 testing by volunteers at the Centre for Genomic Regulation

**DOI:** 10.12688/f1000research.27497.1

**Published:** 2020-11-16

**Authors:** Ritobrata Ghose, Álvaro Aranguren-Ibáñez, Niccolò Arecco, Diego Balboa, Marc Bataller, Sergi Beltran, Hannah Benisty, Angèle Bénard, Edgar Bernardo, Sílvia Carbonell Sala, Eloi Casals, Ludovica Ciampi, Livia Condemi, Alberto Corvó, Marta Cosín-Tomás, Mirabai Cuenca-Ardura, Juan Manuel Duran Serrano, María Isabel Espejo Díaz, Marcos Fernandez Callejo, Antoni Gañez-Zapater, Raquel Garcia-Castellanos, Romina Garrido, Gil Henkin, Toni Hermoso Pulido, Xavier Hernandez-Alias, Jorge Herrero Vicente, Matthew Ingham, Wei Ming Lim, Sílvia Llonch, Elena Marmesat Bertoli, Irene Miguel-Escalada, Ariadna Montero-Blay, Cristina Navarrete Hernández, Maria Victoria Neguembor, Róisín-Ana Ní Chárthaigh, Natalia Pardo-Lorente, Laura Pascual-Reguant, Sílvia Pérez-Lluch, Reyes Perza, Martina Pesaresi, Daniel Picó Amador, Paula Pifarré, Davide Piscia, Marcos Plana-Carmona, Julia Ponomarenko, Leandro Radusky, Ezequiel Rivero, Malgorzata Rogalska, Guillem Torcal Garcia, José Wojnacki

**Affiliations:** 1 Centre for Genomic Regulation (CRG), The Barcelona Institute of Science and Technology, Dr. Aiguader 88, Barcelona, 08003, Spain; 2 Universitat Pompeu Fabra (UPF), Barcelona, 08005, Spain; 3 CNAG‐CRG, Centre for Genomic Regulation, The Barcelona Institute of Science and Technology, Baldiri Reixac 4, Barcelona, 08028, Spain; 4 ISGlobal, Barcelona, 08036, Spain; 5 CIBER de Epidemiología y Salud Pública, Barcelona, 08023, Spain

**Keywords:** COVID-19 testing, volunteers, health, collaboration

## Abstract

The COVID-19 pandemic has posed and is continuously posing enormous societal and health challenges worldwide. The research community has mobilized to develop novel projects to find a cure or a vaccine, as well as to contribute to mass testing, which has been a critical measure to contain the infection in several countries. Through this article, we share our experiences and learnings as a group of volunteers at the Centre for Genomic Regulation (CRG) in Barcelona, Spain. As members of the ORFEU project, an initiative by the Government of Catalonia to achieve mass testing of people at risk and contain the epidemic in Spain, we share our motivations, challenges and the key lessons learnt, which we feel will help better prepare the global society to address similar situations in the future.

## Introduction: CRG in Orpheus’ shoes

The recent turn of events worldwide, brought on by the severe acute respiratory syndrome coronavirus 2 (SARS-CoV-2) that uprooted the world of all normality, presented a tremendous challenge. In tragic unison, COVID-19 became the viper to humanity’s Eurydice, and the world of researchers and medics, her Orpheus, in our attempt to revive and reset. With a name inspired by this mythological allegory, the Catalan government launched the ‘ORFEU’ project – a mass testing initiative to identify infected individuals – in an attempt to reduce infection rates and facilitate an easier transition into a post-confinement era. At the time that the government decided to launch the ORFEU project, the epidemic in Catalonia was at a critical high. In April 2020, the infection rates and mortality were peaking, and the health system was rapidly overwhelmed. As a result, no systematic testing initiatives were in place in various critical settings such as nursing homes, prisons or clinical institutions due to the vast demand for tests for symptomatic people in hospitals.

**Figure 1.**
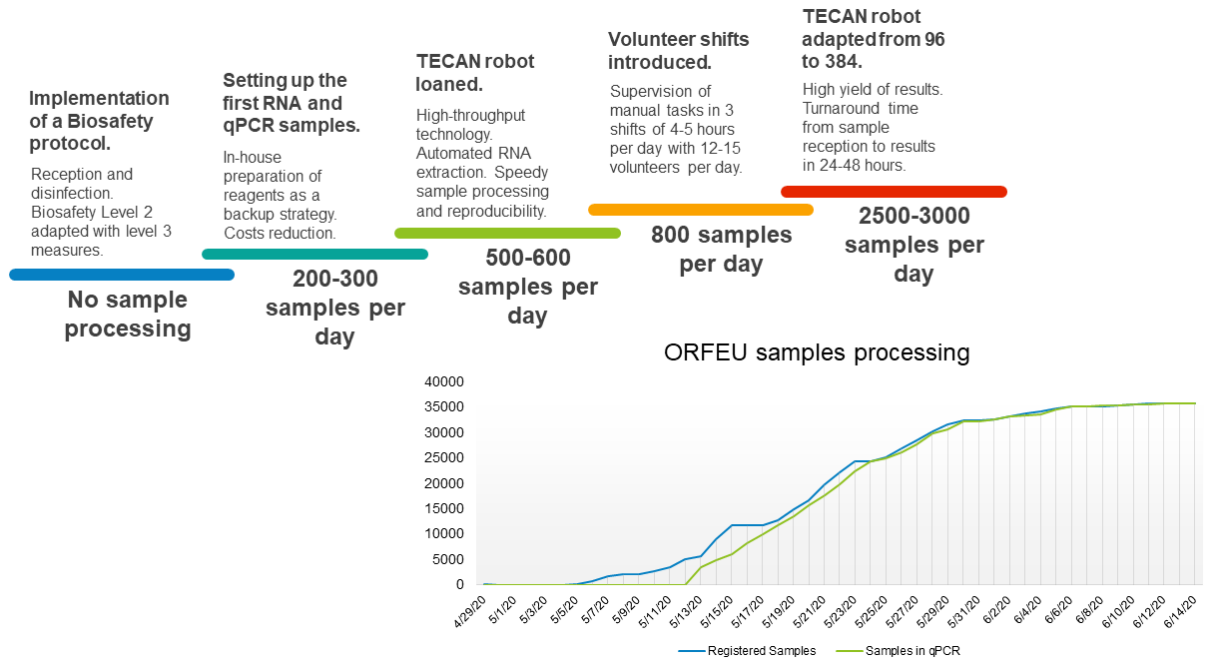
Key milestones towards increasing sampling processing. (Top) Modifications to the workflow, which greatly influenced our ability to handle a larger number of samples. (Bottom) Cumulative number of samples processed from the start to the end of the ORFEU project.

**Figure 2.**
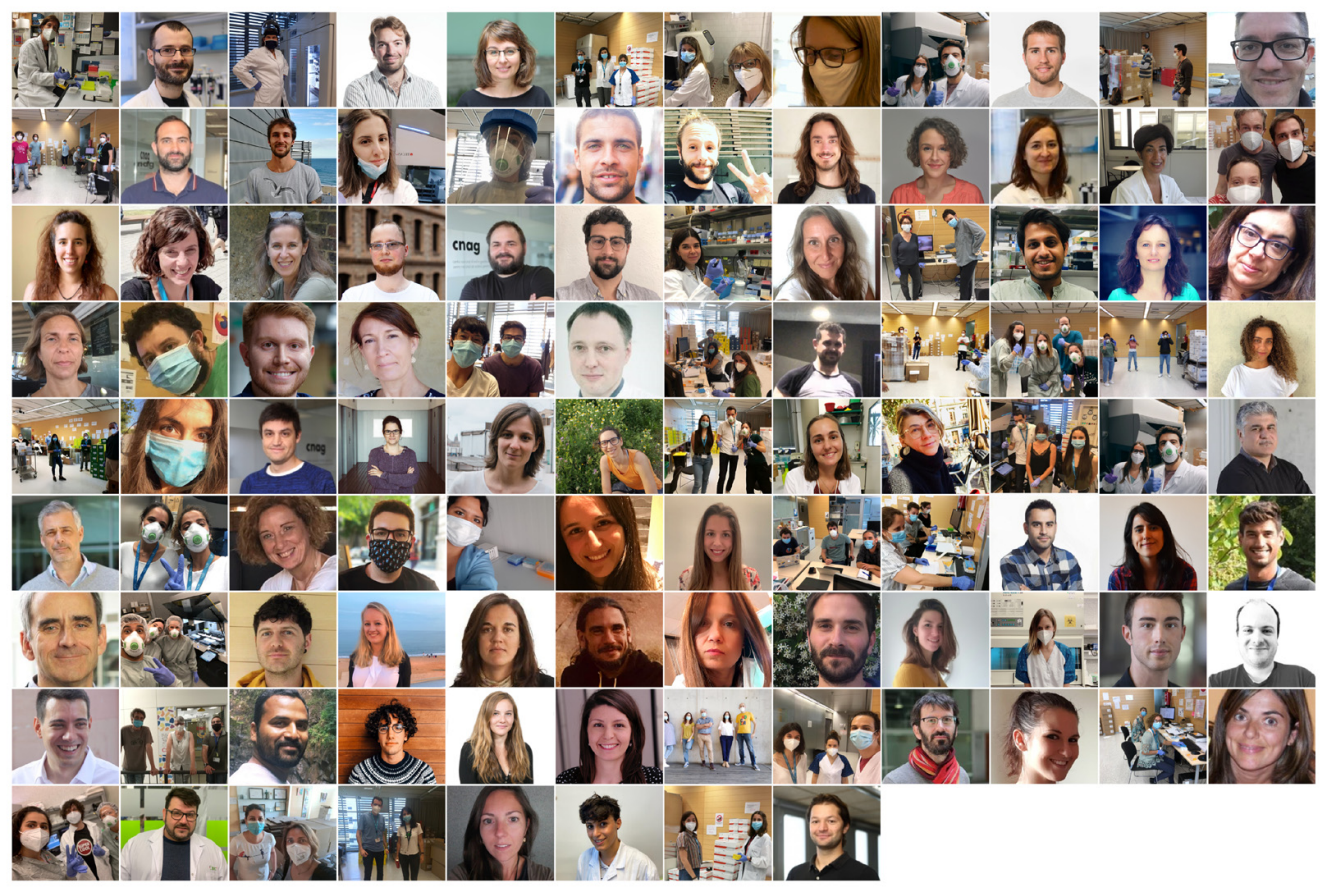
Some of the volunteers across the Barcelona Biomedical Research Park and Barcelona Science Park nodes who took part in the ORFEU project. We confirm that we have obtained written informed consent to use images of the individuals included in this presentation.

With the aim of performing 170,000 PCR-based COVID-19 tests, the ORFEU project united some of the major research institutes across Catalonia. There were two main sample-processing nodes with close to 200 volunteers collectively – one at the Barcelona Biomedical Research Park (PRBB) orchestrated by the Center for Genomic Regulation (CRG) and the other at Barcelona Science Park (PCB), jointly coordinated by IRB Barcelona, IBEC and the CNAG-CRG ( Figure 1). Both nodes were supported by the CNAG-CRG informatics team and the whole process coordinated by the CRG director. The team at PRBB comprised a total of 106 volunteers including principal investigators, post-doctoral researchers, technicians, and administration personnel ( Figure 2). Here, in this short article, we, as volunteers at the PRBB node, share our motivations, and briefly outline the processes and challenges that were important to adapt from a basic research centre to an analytical and diagnostic centre for COVID-19 so that other global institutes may benefit from our experience.

## The motivation

“ *Extraordinary times call for extraordinary measures. We saw a need that needed to be filled and we stepped in to help.*” - Benet Wilson

The motivations of all the volunteers were diverse. While for some it was curiosity to better understand the on-ground scene, others hoped to get a much-desired alternative to in-house confinement. For others less fortunate, the motivation was fuelled by personal experiences with loved ones having suffered through the infection. But most of all, and unanimously, the underlying motivation was to help society fight against the pandemic by offering our specific skill sets and spreading the knowledge needed to battle the crisis. Together, obstacles such as reduced public transport, commuting distances and the inherent human fear factor, were overcome by our will to curb the pandemic through our contributions in the ORFEU project.

## Organisation and pipeline

At the outset, once called into the ORFEU project, the CRG health and safety department achieved the mammoth task of adapting the research centre into an analytical and diagnostic centre. There were two major phases to this. First, a biosafety protocol outlining key information such as SARS-CoV-2 sample collection and handling, based on the procedures from the World Health Organisation ^1^ and Centre for Disease Control and Prevention ^2^ were established. In addition, a Biosafety Level 2 facility was modified by introducing level 3 measures, such as exclusive changing rooms and protective equipment disposal protocols, which was pivotal in our ability to deal with SARS-CoV-2 samples due to the need for adequate disinfection and correct storage. In the second phase, given our inexperience in handling such an emergency, adequate training of volunteers was critical. To start, a core team of volunteers of around 10 members were instructed on biosafety measures through an online training session given by an external expert in Biosafety Level 3 followed by a hands-on *in situ* training in smaller groups. All members were trained equally in sample collection and disinfection, sample registration, RNA extraction, PCR and validation, allowing fail-safes and contingencies in cases when members were not able to carry on for any reason. Eventually more volunteers were recruited based on sample load and divided into specialised groups led by a member from the core team. The entire workflow was monitored using a laboratory information management system (LIMS) developed *ad hoc* by the Bioinformatics Unit at CNAG-CRG and a user interface program for sample registration developed by the CRG bioinformatics unit. Together, the LIMS allowed for the smooth and rapid management of samples in an efficient and errorless process between various teams. Expert clinical microbiologists, from various collaborating hospitals, interpreted the PCR results online in a user-friendly module of the LIMS, and carried out the final task of diagnosis.

The ultimate aim was to automate the process for speedy sample processing as well as reproducibility using high-throughput TECAN robots (one of which was kindly loaned to the CRG by the company) and multiple RT-PCR machines (one kindly lent by the University Pompeu Fabra). Nevertheless, the importance of manual tasks and supervision was paramount, and required approximately three shifts of 4–5 hours every other day, with approximately 4–5 volunteers per shift. Having started at a few hundred samples processed per day, by the end of the project, the CRG was in a position to process 2500–3000 samples per day, with a turnaround time from sample reception to results in about 24–48 hours ( Figure 1). The workflow can be followed in an infographic created by CRG ^3^.

## Key challenges

To ensure the systematic running of the entire unit, it was important to establish a general workflow based on the process mentioned above. Through our implementation of the setup ^4^, we identified key steps to assure process standardization and optimisation. Here we highlight some of the major points.

### From the point of view of the organising committee

A project of this scale requires navigating through a large amount of administrative and legal procedures, as well as coordinating many different organisations and teams. Resolving each of these issues caused an initial delay between the proposed and actual start of the project, resulting in a lower overall turnaround of test results than intended. Moreover, the sporadic receipt of samples from various parts of Catalonia presented a challenge in terms of organising shifts, adequate storage and handling, and quick turnaround time (ideally 24–48h). The CRG now has a go-to model ready for use in future situations to share with other organizations ^4^.

### From the point of view of informaticians

One of the main challenges during the project was the simultaneous development of an online informatics platform, whilst the laboratory workflow was being designed. Our aim was to develop a platform that enabled tracking of all samples throughout the process internally and also provided an interactive and user-friendly analysis environment for third-party use by collaborating microbiologists to interpret the results. To achieve this, the CNAG-CRG, together with CRG bioinformaticians, developed a LIMS that included a diagnosis validation tool. 

### From the point of view of the analytical team

The PCR testing protocol had two major aspects – RNA extraction and real-time quantitative PCR. Identifying the optimal combination of reagents with sample processing equipment was arguably the most challenging part and is a must to be tackled at the earliest. The initial phase was subject to continual switching between different RNA extraction kits and equipment, which called for exhaustively long working hours and time-consuming daily training.

Communication and transfer of knowledge posed major challenges as well. Using dedicated social media channels and live cloud-based services helped in sharing status updates between shifts and quick responses to unexpected technical issues.

### From the point of view of the protein production team

A continuous logistical challenge throughout the course of the project was the limited availability of reagents and materials. In order to circumvent this issue, reduce the pressure on manufacturing units and optimise overall project costs, the CRG organised a protein production team that performed expression and purification of all the necessary proteins as a backup in the event that the supply chain broke completely. Protocols were initially tested on a small scale by the CRG’s Protein Technologies Unit and further expanded by volunteers. Moreover, extensive further validation and certification of these proteins in PCR mixes or as other reagents are limiting steps prior to them being available for analytical use.

### From a personal perspective

Often taken for granted, one of the main challenges during the coordination of a crisis initiative such as the ORFEU project was taking into account personal and professional lives. On one hand, at a personal level, the stress of the lockdown and having children at home or living with high-risk individuals made it difficult to volunteer. Accommodating for lockdown-induced anxiety and mental health needs was very important. On the other hand, from a professional point of view, the delays and late start of the project, which coincided with the reopening of many labs, made it difficult to balance between the two. In addition, and very importantly, as basic researchers, we needed to accept a new responsibility, that results from this project would directly impact people’s lives.

## Success stories

The outcomes we achieved in the ORFEU project would not have been possible without some key aspects that contributed to its success.

- Above all, the motivation, commitment and effort of everyone involved, working towards a common objective, was a very strong driving force that kept us on track, despite the obstacles. The trust of the administration in the volunteers allowed us to take the lead on various aspects of the project, enabling productivity and creating an atmosphere of trust and motivation.
- Teamwork and cooperation were, as always, critical. Working together in a friendly, supportive and cooperative environment made it easier to cope with stress and difficult situations without unnecessary conflict. The level of mutual respect was very high and communication was transparent, preventing any tension within the team despite varying opinions, changing protocols and long working hours.
- While the entire team’s effort was very important, it is necessary to highlight that without the dedication and perseverance of the initial organising team, and the various principal investigators, the project would not have been possible. Their tireless commitment for the optimisation of protocols and round-the-clock availability were indispensable stepping-stones for the rest of the team to be able to deliver on the project targets.
- A major turning point in our ability to handle large volumes of samples came through the tireless efforts of highly skilled personnel at the CRG core facilities who worked to overcome technical hurdles and managed, for instance, to convert a robot that was processing 96 samples every 90 min to processing 384 samples in the same time.
- Delegation of tasks and specialised teams became a critical factor in speeding up not only the sample-processing rate but also the training and induction of different groups of volunteers. Given the clear communication and coordination between specialised teams, typically overseen by rotating team leaders, the whole process adopted a very smooth and high-throughput functioning capacity.

## Lessons learned

***Converting a research centre into an analytical platform is not easy:*** Despite the resources and technical expertise, setting-up an analytical platform from scratch and under a state of emergency was a complex process. Added to the challenges intrinsic to the novel social responsibilities, and the professional responsibility of delivering accurate results, the preparation of this platform required various obstacles to be negotiated.

***Create a collaborative work environment:*** A strong team built through collaboration between researchers, the government and health professionals, with a varied skill set and good morale proved to be essential. Stratified decision-making, accountability, and distribution of tasks can promote the efficient functioning of the unit. Moreover, cross-institute collaborations, which arise from such projects, are long-term connections that can present opportunities for collaboration on various projects across disciplines and sectors, for instance between basic researchers, clinicians, health authorities and policymakers.

***Technical assurance is critical:*** The transition from the creation of LIMS to its usability was an important process requiring optimization and often limiting sample processing rates. During the user interface design, it was critical to avoid making assumptions. The entire development process benefited from monitoring usage of the tool after an initial training session, as well as through the course of the project. Documenting workflows and database schemas was fundamental. Additionally, prioritization and differentiation between critical and non-critical features allowed the optimal use of limited software development personnel.

****Importance of good communication:**** Communication is one of the cornerstones of an efficient system. Establishing communication channels quickly through social media, live cloud-based services or internal institute-based communication systems available to everyone involved was very important to guarantee an effective workflow, and to ensure transparency, robustness and stability.

****Protocol standardization:**** The initial stages of the ORFEU project witnessed a lot of changing protocols and volunteers were required to adapt very quickly. Multiple tests were needed to optimize both processes, and as mentioned above technical developments were needed to increase the capacity of the RNA extraction robots. Although not discussed here, we found numerous artefacts in the PCR reactions. For instance, something as banal as minor traces of ethanol in the reaction could induce artifactual amplification, or the contamination of neighbouring wells in plates where a patient had a very large viral load. See 4 for our final protocol.

****Open access and availability of organizational workflow and protocols:**** All protocols, as well as optimization trials (including negative results), from situations such as the COVID-19 pandemic, would gain from being shared openly by enabling other research centres and institutions to make better use of their resources ^5^. Moreover, these are important opportunities to initiate dialogue and collaboration between local and global research institutions, alongside local governments and health authorities. This would allow them to put in place emergency plans and workflows that can prevent loss of precious time and scarce resources, as was exemplified in the initial phases of the ORFEU project.

***Researchers and research are important to society***: Beyond knowledge creation, researchers can mobilize quickly to produce tangible outcomes for the benefit of society. Proven across Catalonia, and globally, researchers can respond and adapt rapidly and effectively to emergencies, collaborating with local healthcare systems. As a result, a greater consideration for scientific research from governments, health authorities and society would better leverage resources during a health crisis.

## Our top 10 recommendations to be prepared for the next pandemic

1. A general but adaptable workflow should initially be established by a core team of administrators, researchers and informaticians. Additional teams should closely follow to prevent exhaustion of the leading team and sustain productivity. To ensure readiness, an emergency response team should revisit the entire workflow annually and adapt it to address any potential issues based on current infrastructure, availability of personnel, and other possible complications.
2. Define clear tasks and responsibilities as soon as possible. The combination of specialized teams and a small group of people with broader knowledge ensures efficiency and robustness.
3. An initial database schema should be agreed upon ahead of time followed by suitable data transfer formats and protocols between systems (e.g. LIMS and Electronic Health Records). To ensure the systematic transfer of samples and eventually results, set up a common platform with the health authorities to assure transparent communication of all necessary logistic information.
4. Set up dedicated communication channels that allow fast information transfer and updates.
5. Volunteers should be recurrently tested, and not exclusively at the start of the programme. This will ensure mental peace and health safety of all directly or indirectly involved, as a result, ensuring sustainability of the project.
6. Set up a pipeline to produce your own reagents (RT-PCR master mix and RNA extraction kits), so as not to solely rely on external sources to acquire them.
7. Open access protocols should be shared across research centres and health institutions as soon as they are standardised. Problems arising during their development should also be communicated to avoid needless repetition.
8. Equipment and pipelines should not be dismantled until normality has been reinstated globally to prepare for future outbreaks and the possible need for testing protocols.
9. In emergencies, requirements are dynamic and processes can change very quickly. Hence, the volunteer recruitment process should continue as a backup for as long as possible.
10. Despite being volunteer work, all participants worked at their own projects’ expense. Understanding from supervisors and recognition from funding agencies and government bodies, for instance by extending fellowships, would attract more support.

## Data availability

No data are associated with this article.
