# Supplementary material for: From research to rapid response: mass COVID-19 testing by volunteers at the Centre for Genomic Regulation
Source: F1000Res. 2020 Nov 16;9:1336. [Version 1] doi: 10.12688/f1000research.27497.1 (PMC8564741; doi:10.12688/f1000research.27497.1)
Supplement: Peer review history

# Peer review: Reviewer response for version 1

**DOI:** 10.5256/f1000research.30390.r96371

**Published:** 2021-11-02

**Authors:** Umair Ahmed Bargir

**Affiliations:** 1 Department of Pediatric Immunology and Leukocyte Biology, ICMR-National Institute of Immunohaematology, Mumbai, Maharashtra, India

Ghose *et al.*, in this opinion article, highlight the success behind the mammoth responsibility which was imposed upon the Catalan government and the role played by volunteers and researchers of the ORFEU project.

 The authors have highlighted the key challenges faced and the lessons learned while successfully implementing this mammoth task. To overcome similar challenges that can arise in the future, authors have given a ten-point recommendation to be prepared for the subsequent pandemics.

Is the topic of the opinion article discussed accurately in the context of the current literature?

Yes

Are arguments sufficiently supported by evidence from the published literature?

Yes

Are all factual statements correct and adequately supported by citations?

Yes

Are the conclusions drawn balanced and justified on the basis of the presented arguments?

Yes

Reviewer Expertise:

Immunology, Genomics

I confirm that I have read this submission and believe that I have an appropriate level of expertise to confirm that it is of an acceptable scientific standard.

# Peer review: Reviewer response for version 1

**DOI:** 10.5256/f1000research.30390.r74857

**Published:** 2020-12-16

**Authors:** Ricardo Leite

**Affiliations:** 1 Instituto Gulbenkian de Ciência, Oeiras, Portugal

This opinion article is truly an example on how scientists have assisted society during COVID-19 pandemic, by adapting and perform wide-scale testing in their communities. Scientists have a set of skills and knowledge of protocols, techniques and methodology, and these resources are sometimes ignored and left out by health authorities. That was not the case of Catalan government decided to launch ORFEU program, to avail the existing expertise and test capability and scale it up with the help of many volunteers that mobilize to perform a massive effort of COVID-19 testing. This article documents the insight vision, point of view, motivation and challenges of the volunteers from PRBB during this process.

 Besides the success stories, the learned lessons and perspectives from this transition and this period are very important and they can be explored in less troubled times.

 It is important to pinpoint some of these lessons such as: balancing mental health and anxiety with new responsibilities; creating a collaborative work environment based on trust and cross institutes collaborations; good communication and mechanisms for sharing open data (protocols, workflows) and the effort on standardization and automatization of protocols. Although not fully discussed, the protocols and guidelines are referenced along the text and are available for consultation.

 The ten recommendations are clear and solid and undoubtedly resulting from an intensive planification effort and posterior discussion and it can be directly transferred to other institutes, to be consider in new upcoming events and future outbreaks.

Is the topic of the opinion article discussed accurately in the context of the current literature?

Yes

Are arguments sufficiently supported by evidence from the published literature?

Yes

Are all factual statements correct and adequately supported by citations?

Yes

Are the conclusions drawn balanced and justified on the basis of the presented arguments?

Yes

Reviewer Expertise:

Genomics, sequencing, single cell, bioinformatics.

I confirm that I have read this submission and believe that I have an appropriate level of expertise to confirm that it is of an acceptable scientific standard.
